# Perirhinal cortex lesions that impair object recognition memory spare landmark discriminations

**DOI:** 10.1016/j.bbr.2016.07.031

**Published:** 2016-10-15

**Authors:** Andrew J.D. Nelson, Cristian M. Olarte-Sánchez, Eman Amin, John P. Aggleton

**Affiliations:** School of Psychology, Cardiff University, 70 Park Place, Cardiff, CF10 3AT, Wales, UK

**Keywords:** Hippocampus, Navigation, Parahippocampal cortex

## Abstract

Rats with lesions in the perirhinal cortex and their control group learnt to discriminate between mirror-imaged visual landmarks to find a submerged platform in a watermaze. Rats initially learnt this discrimination passively, in that they were repeatedly placed on the platform in one corner of a square watermaze with walls of different appearance, prior to swimming to that same location for the first time in a subsequent probe trial. Perirhinal cortex lesions spared this passively learnt ability, despite the common visual elements shared by the guiding landmarks. These results challenge models of perirhinal function that emphasise its role in solving discriminations between stimuli with ambiguous or overlapping features, while underlining how this cortical region is often not required for spatial processes that involve the hippocampus.

## Highlights

• Loss of perirhinal cortex spares mirror-imaged landmark discriminations.
• Perirhinal cortex lesions do not disrupt latent spatial learning.
• Further underlines dissociation between perirhinal and hippocampal function.

There is compelling evidence that perirhinal cortex damage disrupts tests of object recognition [1], [2]. This region is also involved in ‘object-in-place’ memory, which taxes the ability to remember the location in which a particular object was encountered [3], [4]. Despite being heavily interconnected, the perirhinal cortex and hippocampus appear to play rather different roles in memory [5]. For example, perirhinal cortex lesions often spare spatial tasks that are highly sensitive to hippocampal damage. One influential account of perirhinal cortex function holds that the perirhinal cortex represents conjunctions of stimulus features and so aids discriminations between stimuli with overlapping features [6]. A prediction that follows from this account is that perirhinal damage should increase sensitivity to interference from related stimuli that share common features. Evidence in support of this position comes from studies showing that perirhinal cortex lesions can impair visual discriminations involving feature ambiguity [7], [8], [9], [10]. This account can also readily explain why perirhinal lesions can often spare performance on spatial memory tasks, as perirhinal lesions might only be expected to disrupt those tests of spatial memory that involve discriminations between stimuli with overlapping or ambiguous visual features.

**Fig. 1:**
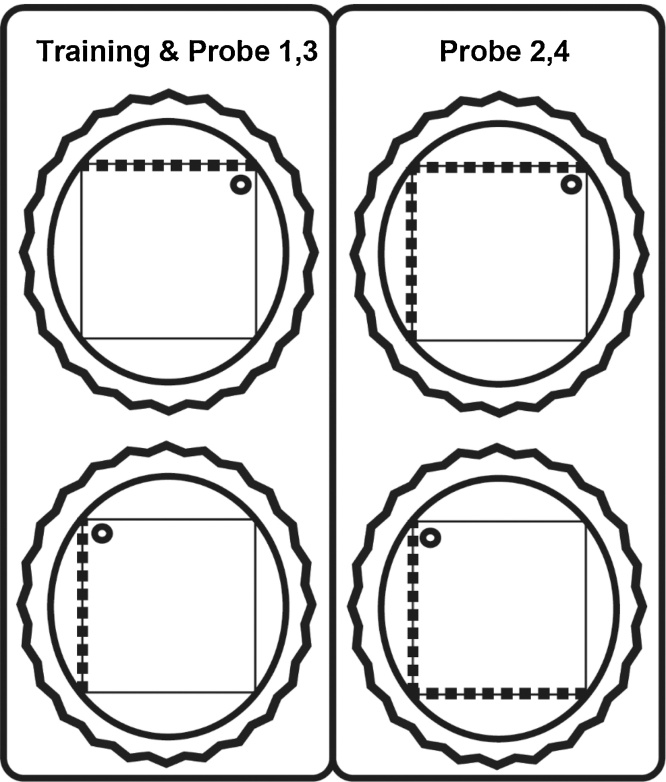
Schematic diagram of the square maze with different patterned walls. The inner square shape depicts the pool, the surrounding circle is the larger pool within which the square pool is placed, and the rippled circle represents the curtain used to block distal cues. The broken lines represent striped walls. The small circle represents the platform on which the rat was placed (passive trials). In Probes 1 & 3 (left column), the maze had one striped wall, as was the case in all active and passive training days. In Probes 2 & 4 (right column), the maze had two adjacent striped walls. ‘Passive’ refers to training days when the rat was placed on the escape platform (no swimming). ‘Active’ refers to training days when the rat was placed in the water-maze and swam to find the escape platform.

The present study examined the impact of perirhinal cortex lesions on the ability to discriminate mirror-imaged landmarks in order to find a submerged platform in a square water-maze. Mirror-imaged stimuli are of especial interest as they share the same elements and, hence, should be prone to interference. Although a previous study indicated that perirhinal lesions spare mirror-image discriminations [11], the training protocol was very protracted. The present study differed in two key aspects. First, it used a more rapid learning protocol [12], [13]. Second, learning was ‘passive’ in that there was no explicit reinforcement for learning the discrimination. Accordingly, rats were repeatedly placed on a partially submerged escape platform in the corner of a square water-maze where the platform location was signalled by the spatial arrangement of mirror-imaged patterns on adjacent maze walls. The particular discrimination involved those corners where striped walls and white walls met, where their relative left/right position was critical (see Fig. 1). Normal rats then swim to the escape location when first released into the water after passive training, so demonstrating their discrimination between these visual landmarks. As the animals have no experience of swimming to find the escape platform in this location prior to the probe test, this problem can only be solved by learning the correct configuration of spatial cues, rather than by simpler mediating strategies involving individual stimuli (e.g. swim to the striped wall and turn right, see Fig. 1) that could be acquired during active training [12]. The key question was whether perirhinal cortex damage would disrupts rats’ ability to identify the correct configuration given the presence of common cues with shared features.

Twenty-nine male Lister-Hooded rats (Harlan, Bicester, UK) were used. All experiments were in accordance with the UK Animals (Scientific Procedures) Act (1986) as well as EU directive 2010/63/EU. Surgical procedures and care proceeded as described previously [23]). Injections of 0.09 M *N*-methyl-d-aspartic acid (NMDA; Sigma, Poole, U.K.) dissolved in phosphate-buffered saline (pH 7.4) were made in three sites using a 26 gauge, 1-μl Hamilton syringe (Bonaduz, Switzerland). The injection coordinates relative to bregma and volume of NDMA infused were (1) AP −1.8, ML ±5.9, DV −9.3 (0.22 μl); (2) AP −3.4, ML ±6.2, DV −9.5 (0.20 μl); (3) AP −5.0, ML ±6.3, DV −8.9 (0.20 μl). Injections were at a rate of 0.10 ml per min, with the needle left in place for four minutes after each injection.

**Fig. 2:**
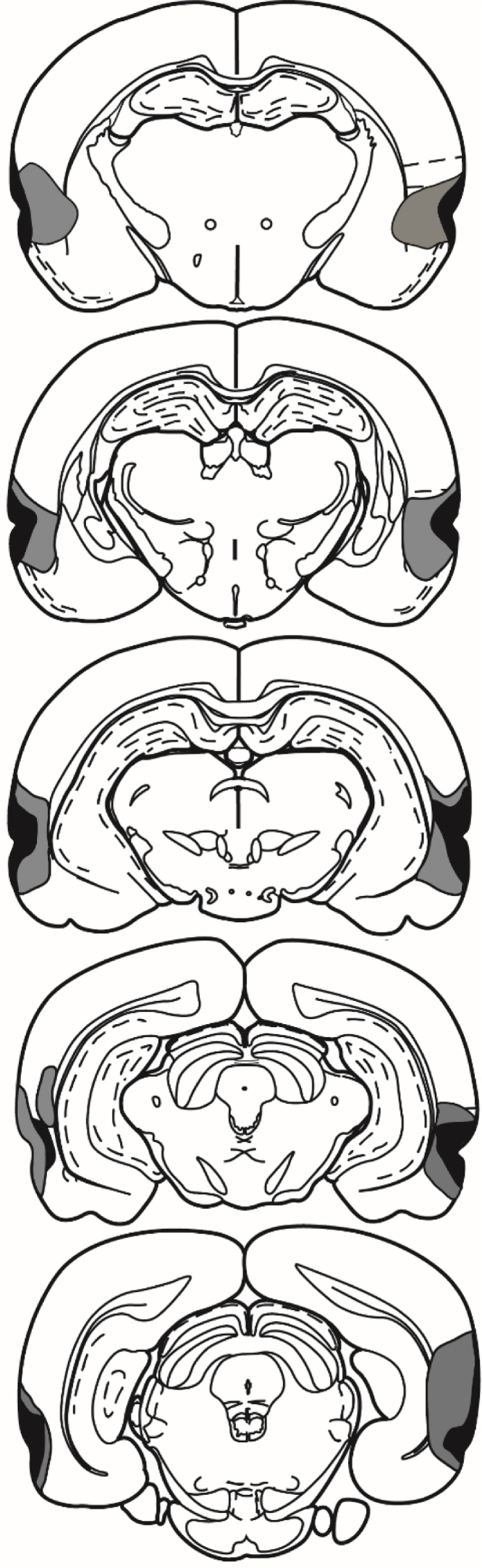
Diagrammatic reconstructions of the perirhinal cortex lesions, showing the individual cases with the largest (grey) and smallest (black) lesions. The most rostral coronal section is at the top. The sections are ∼1 mm apart in the AP plane.

The lesions, consistently centred on the rhinal sulcus, caused extensive bilateral damage to areas 35 and 36 (Fig. 2). One case was rejected because of bilateral sparing, leaving 16 perirhinal and 12 sham animals. The lesions involved almost the full anterior-posterior extent of areas 35 and 36. The mean percentage of perirhinal cortex loss was 76.0% (range 53.7–95.0%). A frequent feature was some cell loss in the adjacent part of the piriform cortex and lateral entorhinal cortex. There was often limited damage to that part of CA1 immediately adjacent of the fundus of the rhinal sulcus (7 unilateral, 7 bilateral) but was typically very restricted to the level of the most caudal rhinal sulcus.

The apparatus and procedure matched those used to analyse hippocampal lesions [14]. Briefly, rats began with four days of pre-training (four trials a day) during which rats swam in a circular pool (2 m diameter) to find a submerged platform. Pre-training took place in a different maze and room to subsequent training. By pre-training day 4, there was no group difference in latency to reach the platform (*t* *<* *1*, means Perirhinal = 59.3, Sham = 56.5 s).

Next, the rats were trained passively in the square pool with the platform in a fixed position with respect to the corners. The pool (140 cm × 140 cm) was set within a larger white, circular tank, measuring 2.0 m in diameter and 60 cm deep (Fig. 1). The walls of the square-shaped pool were formed by one black and white striped and three white Perspex boards (140 cm long, 50 cm high, and 2 mm thick). The vertical black stripes were 10 cm wide with 10 cm white intervals. The black stripes began 5 cm from the side edge of the board. The maze configuration created three sets of corners: (1) black and white striped wall to the left of the white wall, (2) black and white striped wall to the right of the white wall, and (3) white wall meeting white wall (two corners; see Fig. 1). An escape platform (10 cm in diameter) was submerged 2 cm below the water surface. To occlude room cues, a curtain was drawn around the pool.

**Fig. 3:**
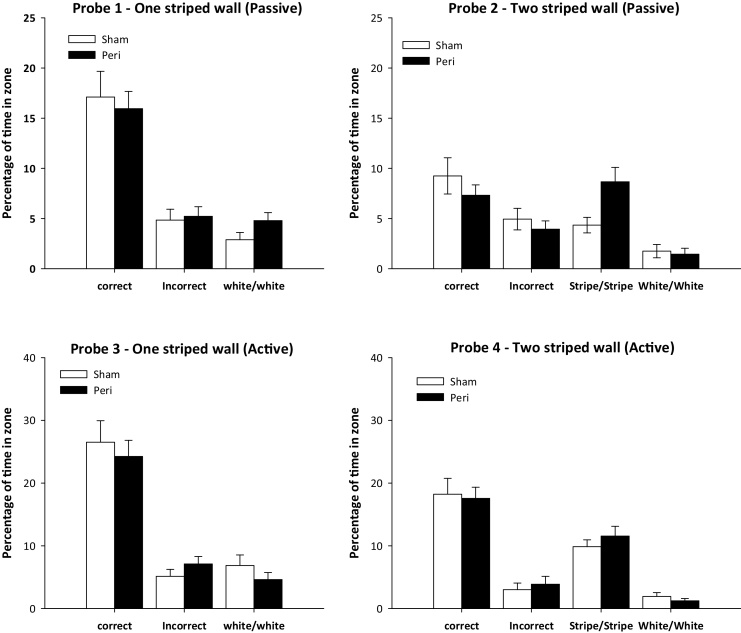
Passive learning of a corner location in the square water-maze. Each graph shows the proportion of time spent in the trained (‘Correct’) corner of the maze. One corner was the mirror-image of the training corner (‘Incorrect’). 13. In Probes 1 and 3 the maze contained three white walls and one striped wall. In Probes 2 and 4 the maze contained two adjacent white walls and two adjacent striped walls. Probes 1 and 2 followed ‘passive’ training while Probes 3 and 4 followed ‘active’ training.

The rats were placed on the platform for eight days (four trials a day). The platform was positioned 25 cm from a corner on an imaginary line that bisected the corner. This position was counterbalanced, so that half of the rats had the platform placed in a corner where the striped wall was to the right of a white wall. For the other half, the platform was in the corner where the striped wall was to the left of the white wall. To nullify any extraneous cues and ensure that the task could only be solved by discriminating between the mirror-imaged landmarks, the square pool was randomly rotated 90°, 180°, or 270° clockwise between each trial. Rats were placed individually on the escape platform for 30 s on four separate trials (inter-trial interval approximately four minutes). On day eight, the rats received three training trials followed by a test trial (Probe 1, ‘One striped wall’). For Probe 1, the platform was removed, the rats placed in the centre of the square pool, and allowed 60 s to swim in the water for the first time. The latencies to reach the escape location did not differ between the two groups (*t* < 1; means, Perirhinal = 20.9s, Sham = 24.7 s). There were no group difference when the times in the correct corner (*t* < 1), incorrect corner (*t* < 1), or white:white corner (*t_26_* = 1,17, *P* = 0.099) were separately compared between the two groups (Fig. 3).

Next, the rats were placed on the escape platform for an additional session (four passive trials) with one striped pool wall. The following day, the first three trials involved standard passive training. The fourth trial was Probe 2, ‘Two striped walls’. Now the pool had two black and white striped walls arranged next to each other for the first time (Fig. 2). On this second probe trial, the rat was again put into the water in the centre of the pool and allowed to swim for 60 s (platform absent). The ‘correct’ and ‘incorrect’ corners, along with the white:white corner, remained, but a new incorrect corner (‘novel’) composed of the meeting of two striped walls was created (Fig. 1). There was no group difference (*t* < 1) in the time to first reach the escape location (means, Perirhinal = 38.7 s, Sham = 29.8 s). While there were no group differences for the times spent in the correct, incorrect, or white:white corners (all *t* < 1), the Perirhinal group showed a greater preference for the novel striped:striped corner than the Sham group (*t_26_* = 2.40, *P* = 0.024; Fig. 3).

**Fig. 4:**
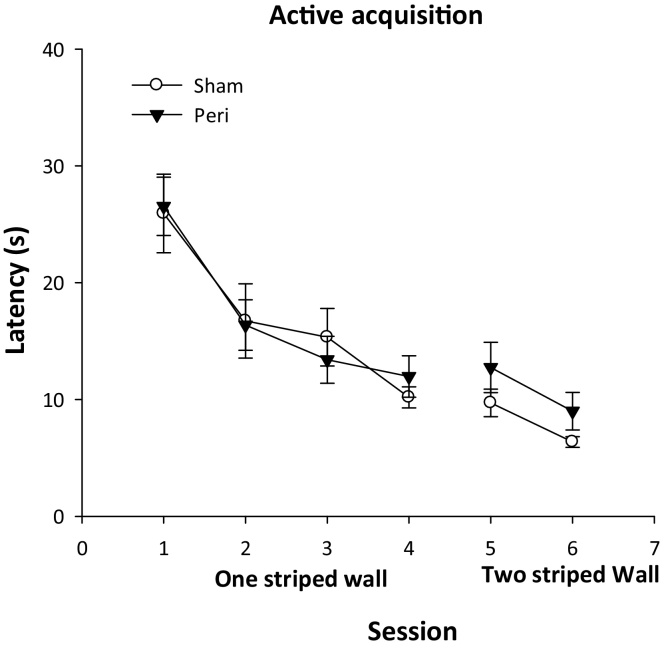
Active learning of the location of an escape platform in a corner of the water-maze. The graphs show the latency to reach the platform when released in the centre of the pool.

The animals then received four sessions (four trials per session) of active training in the square pool with one striped wall (Fig. 1). On each trial, rats were required to escape from the pool by swimming to the submerged platform located in the same corner as during passive training. The rat was placed in the centre of the pool facing the middle of one of the walls. Rats were allowed 60 s to locate the submerged platform. Rats remained on the platform for 30s. Between each trial, the square pool was rotated either clockwise or anticlockwise 90°. The mean escape latency (Fig. 4) decreased with training (*F_3,78_* = 27.7, *P* < 0.001), with no group effect (*F* < 1) nor interaction (*F* < 1).

The last trial of Session four involved a further probe test in the square pool (Probe 3–One striped wall). The first three trials proceeded as above. The platform was then removed and the rat released in the centre of the pool. Each rat was allowed to swim for 60 s in the square maze containing one striped wall (Fig. 3). There was no group difference in the times to reach the escape location (*t_26_* = 1.32, *P* = 0.20; means, Perirhinal = 15.2 s, Sham = 10.0 s). There were no group differences in the time spent in the correct (t < 1), incorrect (*t_26_* = 1.18, *P* = 0.25), or white:white (*t_26_* = 1.16, *P* = 0.26) corners.

In Session five, the rats completed four more swim trials with the one striped wall. In Session six, the platform was removed for the final trial. On this fourth trial, the single striped wall was replaced with two, adjacent striped walls (Probe 4, ‘Two striped walls’). The rat was allowed to swim for 60 s after being released from the centre of the pool. There was no group difference in the latency to reach the escape location (*t_26_* = 1.16, *P* = 0.26; means Perirhinal = 9.0 s, Sham = 6.6 s). There were no group differences in the times spent in each of the four different corners (largest *t_26_* = 1.05, *P* = 0.31). Thus, unlike the previous probe with two striped walls, there was no indication that the perirhinal lesion group had an excessive preference for the novel corner formed by the meeting of the two striped walls (Fig. 3).

The present study examined passive landmark learning after perirhinal cortex lesions. The procedure required the rat to navigate according to the spatial disposition of a prescribed set of maze cues [12], [13]. In contrast to rats with hippocampal lesions [14], the rats with perirhinal lesions learnt a specific location that required discriminating two mirror-imaged corners (Fig. 1). This finding is consistent with previous evidence that the perirhinal cortex is not essential for spatial discriminations involving ambiguous cues with overlapping features [11], [15]. That perirhinal damage spared latent spatial learning also accords with evidence that the lateral entorhinal, which connects the perirhinal cortex with the hippocampus, is similarly not required for latent spatial learning [16]. More broadly, this sparing accords with the wider finding that perirhinal cortex lesions often spare spatial tasks that are highly sensitive to hippocampal damage [5], [17], [18], [19], [20], [21], [22]. Despite this spared ability to discriminate mirror imaged visual stimuli, these same perirhinal lesion animals were severely impaired on tests of spontaneous object recognition [23].

Subsequent probe tests varied the numbers of striped walls to help determine if the rats had just learnt local features of the maze or were sensitive to the global layout of the square swim-maze, i.e., treated it as though it were a new maze when an additional striped wall was added [11][see 11]. In one of the two probe tests the perirhinal lesions appeared to accentuate the attraction found in normal rats to the novel conjunction of two striped walls [13]. At the same time, the preserved ability to distinguish the correct from the incorrect (mirror-imaged) corners in the various probe tests suggests that both the Perirhinal and Sham rats had learnt local features of the pool [11][see also 11]. Finally, the perirhinal cortex lesions appeared to be without effect when the rats were reinforced for swimming to the platform location to escape, i.e., with active training. Overall, these null results differ from those of hippocampal, retrosplenial cortex, and anterior thalamic nuclei lesions, all of which disrupt the learning of preference for the correct corner of the pool after passive training [14], [24], [25]. This dissociation further highlights differences between the perirhinal cortex and the extended hippocampal system [26].

The finding that rats with perirhinal cortex lesions could discriminate mirror-imaged stimuli suggests that only particular kinds of visual stimuli with common elements are sensitive to perirhinal damage. One explanation, consistent with the hierarchical model [6], would be that the water-maze task involved only simple features (striped vs. white), so sparing the need for the perirhinal cortex. Such stimuli are likely to be processed early in the visual system and so may reach the extended hippocampal system via structures other than the perirhinal cortex. One possible route for this information to reach the hippocampus is via the retrosplenial cortex, damage to which is known to disrupt the current task [25]. A further possibility is that the binding of landmark features and spatial information is processed by the dendate gyrus [27], so that the perirhinal cortex is not required for the current task. A challenge for future studies will be to determine what kinds of ambiguous stimuli are sufficiently complex to be engaged by perirhinal cortex.
